# Early COVID-19 vaccine effectiveness of XBB.1.5 vaccine against hospitalisation and admission to intensive care, the Netherlands, 9 October to 5 December 2023

**DOI:** 10.2807/1560-7917.ES.2024.29.1.2300703

**Published:** 2024-01-04

**Authors:** C Henri van Werkhoven, Anne-Wil Valk, Bente Smagge, Hester E de Melker, Mirjam J Knol, Susan JM Hahné, Susan van den Hof, Brechje de Gier

**Affiliations:** 1 Center for Infectious Disease Control, National Institute for Public Health and the Environment (RIVM), Bilthoven, the Netherlands; 2 Julius Center for Health Sciences and Primary Care, University Medical Center Utrecht, Utrecht, the Netherlands

**Keywords:** COVID-19, Vaccine effectiveness, COVID-19 vaccination

## Abstract

We present early vaccine effectiveness (VE) estimates of the 2023 seasonal COVID-19 XBB.1.5 vaccine against COVID-19 hospitalisation and admission to an intensive care unit (ICU) in previously vaccinated adults ≥ 60 years in the Netherlands. We compared vaccination status of 2,050 hospitalisations including 92 ICU admissions with age group-, sex-, region- and date-specific population vaccination coverage between 9 October and 5 December 2023. VE against hospitalisation was 70.7% (95% CI: 66.6–74.3), VE against ICU admission was 73.3% (95% CI: 42.2–87.6).

In the Netherlands, the 2023 seasonal COVID-19 vaccination campaign started on 2 October, targeting persons aged 60 years and older, healthcare workers, pregnant women and medical risk groups. It used the monovalent XBB.1.5 Comirnaty vaccine (Pfizer-BioNTech). All residents of the Netherlands aged 60 years and older received a personal invitation for vaccination by post between 2 October and the end of November. Up to 17 December 2023, 48.2% of residents aged 60 and older had received the 2023 seasonal COVID-19 vaccination [1]. The seasonal vaccination campaign ran until 22 December 2023. 

Since October 2023, the severe acute respiratory syndrome coronavirus 2 (SARS-CoV-2) RNA load in sewage water has been rising steadily up to the highest level since start of this surveillance system in 2020, indicating a high level of SARS-CoV-2 transmission during the past months [2]. To inform the public and policymakers, we here provide an early estimate of the vaccine effectiveness (VE) of the 2023 seasonal vaccination campaign against COVID-19 hospitalisation and against admission to an intensive care unit (ICU).

## Data sources 

We used the screening method to estimate the 2023 seasonal VE among persons aged 60 years and older (birth year 1962 or before) with at least one previous COVID-19 vaccination who were included in the population register of the Netherlands on 25 September 2023. We extracted hospitalisations with admission dates between 9 October and 5 December 2023 from the National Intensive Care Evaluation (NICE) COVID-19 database on 11 December 2023, to account for registration delay. This dataset contained around 55% of all COVID-19 hospitalisations during the study period in the Netherlands, based on anonymous data on all COVID-19 hospitalisations as reported by the National Coordination Centre for Patient Distribution [3]. 

The NICE COVID-19 database contains a column on the reason for admission. However, the reason for admission was missing for 1,002 of 2,392 (42%) of admissions during the study period. Of 1,390 admissions with known reason for intake, 342 (25%) were excluded because the reason for admission was not related to COVID-19. Because of the missing data, it is possible that SARS-CoV-2 infection was present but was not the reason for admission in a fraction of included admissions. Policies on screening admitted patients for SARS-CoV-2 can vary per hospital and over time.

Hospitalisation data were linked deterministically based on citizen service number to the national COVID-19 vaccination database (CIMS). COVID-19 vaccinations are registered in CIMS when vaccinees provide informed consent for registration. The percentage of persons vaccinated at the Municipal Health Services consenting to registration in CIMS was ≥ 95% during previous booster campaigns, and 98% for the 2023 seasonal campaign [1]. We included only persons with at least one previous vaccination registered in CIMS since January 2021, based on the assumption that this population is most likely to consent to registration of 2023 seasonal vaccination as well, thereby minimising misclassification [4]. 

While the campaign formally started on 2 October 2023, some facilities started vaccination on 25 September. Therefore, we included vaccinations from 25 September onward as 2023 seasonal doses. Persons who received a COVID-19 vaccination in the 90 days before 25 September were excluded from the analysis, because they were not eligible for vaccination at the start of the seasonal campaign per the guideline [5]. To account for the delay in immunological response after vaccination, a person’s vaccination status changed to ‘seasonal dose received’ 7 days after administration of the dose. The 7 days between vaccination and the status ‘seasonal dose received’ were excluded from the analysis. Vaccination status of the cases on the date of admission was determined in the same way. The CIMS registry was used to calculate the proportion of the population with seasonal dose received, stratified by the following covariates: calendar date, sex (male, female), geographical region (25 levels) and 5-year age group. Persons contributed to the proportion of the population vaccinated until the 15th of the month of death or emigration or end of the study period.

The VE and 95% confidence intervals (95% CI) were estimated using a logistic regression model, with vaccination status as dependent variable and the covariate-specific logit of seasonal dose received in the population as offset. The exponentiated intercept of this model was interpreted as the relative risk (RR), and VE was calculated as (1−RR) × 100% [6].

## Vaccine effectiveness estimates

**Figure 1:**
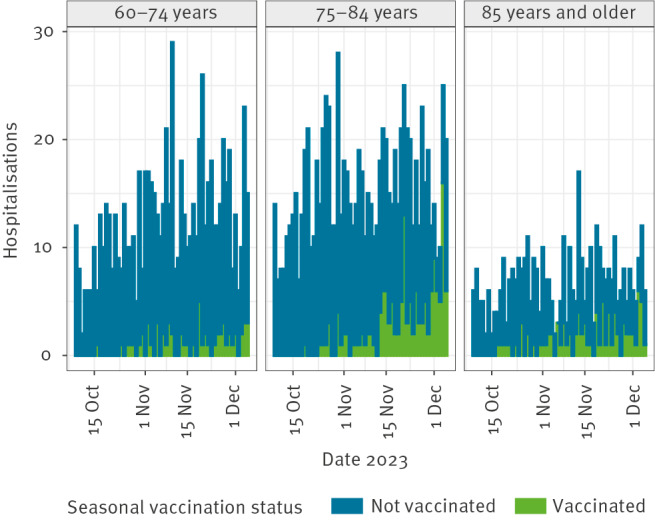
COVID-19 hospitalisations included in the analysis, the Netherlands, 9 October–5 December 2023 (n = 2,050)

**Figure 2:**
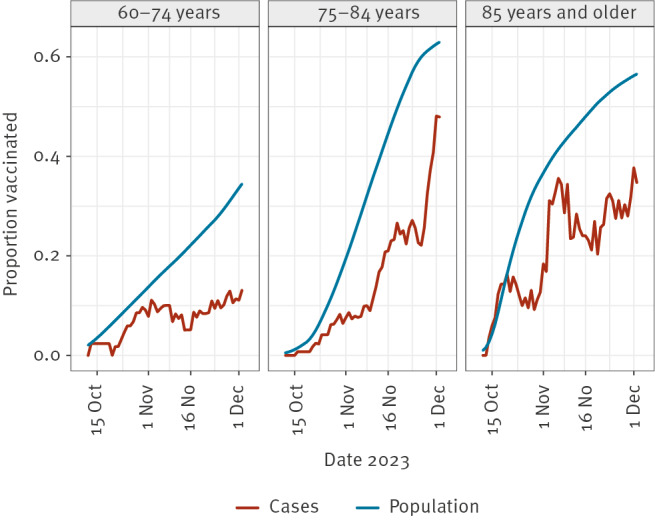
7-day moving average of the proportion of seasonal vaccinees among hospitalised cases and among the population ≥ 60 years eligible for seasonal COVID-19 vaccination on 25 September 2023, the Netherlands, 9 October–5 December 2023 (n = 4,184,231)

After exclusion of 86 hospitalisations (including three ICU admissions) occurring in the 7 days after administration of the seasonal dose, we included 2,050 hospitalised persons, of whom 295 (14.4%) had received the 2023 seasonal COVID-19 vaccination (Table). The number of hospitalisations was higher among persons aged 75–84 years compared with persons aged 60–74 years or 85 years and older (Figure 1). Towards the end of the study period, the proportion of vaccinated increased among both cases and the population, reflecting the gradual roll-out of the campaign (Figure 2). The VE against hospitalisation was estimated at 70.7% (95% CI: 66.6–74.3) (Table). Of the included hospitalisations, 92 concerned ICU admissions, and VE against ICU admission was estimated at 73.3% (95% CI: 42.2–87.6). The VE against hospitalisation estimate was slightly lower for the age groups 60–74 years (68.3%; 95% CI: 58.3–75.9) and 85 years and older (66.0%; 95% CI: 56.4–73.5) than for persons aged 75 to 84 years (73.9%; 95% CI: 68.5–78.4), but these differences were not statistically significant. In the previous year, for the period 3 October 2022 to 12 December 2022, we estimated a slightly lower VE of 64% (95% CI: 59–68) of bivalent booster vaccination against hospitalisation for the age group 60–79 years [7]. However, a direct comparison between these seasonal estimates could be confounded by the fact that the 2022 autumn campaign was closer in time to the previous campaign in spring 2022. 

**Table:** COVID-19 hospitalisations and ICU admissions included in the analysis by seasonal vaccination status, and estimated vaccine effectiveness, the Netherlands, 9 October–5 December 2023 (n = 2,050)

Outcome	Age group (years)	Number of cases with 2023 seasonal vaccination	Number of cases without 2023 seasonal vaccination	VE (95% CI)
**COVID-19 hospitalisation**	≥ 60	295	1,755	70.7% (66.6–74.3)
	60–74	59	681	68.3% (58.3–75.9)
	75–84	150	756	73.9% (68.5–78.4)
	≥ 85	86	318	66.0% (56.4–73.5)
**COVID-19 ICU admission**	≥ 60	8	84	73.3% (42.2–87.6)

CI: confidence interval; ICU: intensive care unit; VE: vaccine effectiveness.

The VE is adjusted for calendar date, region (25 levels), sex and 5-year age group.

Persons with a very high risk of severe COVID-19 could be referred by their medical specialist for an extra vaccine dose in spring 2023. For this group, the 2023 seasonal campaign constituted the fifth booster dose after primary vaccination. However, for only 0.2% of the population aged 60 and older having received the 2023 seasonal dose this constituted a fifth booster. For the majority (76%), the 2023 seasonal dose was the fourth booster dose. On 24 September 2023, the median time since the previous booster dose was 50 weeks, with an interquartile range (IQR) of 48–52 weeks. The number of previous booster doses was lower among the study population who had not (yet) received the 2023 seasonal vaccine (33% one, 27% two and 24% three previous boosters) and on 24 September 2023, their median time since the last booster dose was 61 weeks (IQR: 49–90).

## Discussion

We used the screening method, which we deemed more appropriate than a cohort approach because only a proportion of cases is known. While the NICE hospitalisation database was complete in previous years, this is no longer the case as, due to registration burden, not all hospitals still contribute data. To adjust for possible geographical differences in completeness of hospitalisation data, we included region as a covariate in our model.

A number of limitations may have led to an underestimation of the VE. We were not able to adjust the estimates for comorbidities; such an adjustment generally leads to a higher VE estimate [8]. A study from Denmark among persons aged 65 and older adjusting for comorbidities reported an early 2023 VE estimate against COVID-19 hospitalisation of 75.3% between 8 and 26 October [9]. As our VE estimate is based on the first period of the seasonal campaign, especially the beginning of the study period will represent the VE among early vaccinees, where frail persons could be overrepresented. Further, we defined the vaccination status of cases on the date of hospitalisation, which will be some days after infection and disease progression. Because vaccination coverage was quickly changing during our study period, a relevant proportion of cases was recently vaccinated and might have lacked time for the vaccine to prevent severe disease. If we assessed vaccination status of cases 7 days before hospital admission, the VE estimate would be 74% (95% CI: 69.6–77.7) against hospitalisation for ages 60 and older.

Conversely, our VE might be biased upward due to better general health of seasonal vaccinees compared with the reference group (healthy vaccinee bias). In the Danish study, a hazard ratio of 0.851 was found for hospitalisations for other causes than COVID-19 [9]. In a previous study by our group on VE against COVID-19 mortality in 2021, we found a VE > 50% after primary and first booster vaccination against all-cause mortality, indicating a likely healthy vaccinee bias [8]. Further, if the higher number of doses and/or the shorter time since the last vaccination in the seasonally vaccinated group still conferred some protection during our study period, this may have led to an overestimation of the VE. However, a study among six European countries found that the number of booster doses did not have a relevant effect on VE against hospitalisation, and VE had generally waned 6 months after any booster dose [10].

## Conclusion

This early estimate suggests a high VE against hospitalisation and ICU admission in the first 2 months of seasonal COVID-19 vaccination with XBB.1.5 vaccine. The VE is expected to decrease as the time since vaccination increases in the coming months, as observed in previous COVID-19 vaccination campaigns in Europe. Based on our results, we expect that seasonal COVID-19 vaccination is an effective intervention for reducing the burden of severe COVID-19 during the winter months. An increase in uptake of the seasonal vaccine could further enhance its public health benefit.
